# Balanced Gene Losses, Duplications and Intensive Rearrangements Led to an Unusual Regularly Sized Genome in *Arbutus unedo* Chloroplasts

**DOI:** 10.1371/journal.pone.0079685

**Published:** 2013-11-18

**Authors:** Fernando Martínez-Alberola, Eva M. del Campo, David Lázaro-Gimeno, Sergio Mezquita-Claramonte, Arantxa Molins, Isabel Mateu-Andrés, Joan Pedrola-Monfort, Leonardo M. Casano, Eva Barreno

**Affiliations:** 1 ICBIBE, Departamento de Botánica, Facultad de Ciencias Biológicas, Universitat de València, Burjassot, Valencia, Spain; 2 Departamento de Ciencias de la Vida, Facultad de Biología, Ciencias Ambientales y Química, Universidad de Alcalá, Madrid, Spain; CNR, Italy

## Abstract

Completely sequenced plastomes provide a valuable source of information about the duplication, loss, and transfer events of chloroplast genes and phylogenetic data for resolving relationships among major groups of plants. Moreover, they can also be useful for exploiting chloroplast genetic engineering technology. Ericales account for approximately six per cent of eudicot diversity with 11,545 species from which only three complete plastome sequences are currently available. With the aim of increasing the number of ericalean complete plastome sequences, and to open new perspectives in understanding Mediterranean plant adaptations, a genomic study on the basis of the complete chloroplast genome sequencing of *Arbutus unedo* and an updated phylogenomic analysis of Asteridae was implemented. The chloroplast genome of *A. unedo* shows extensive rearrangements but a medium size (150,897 nt) in comparison to most of angiosperms. A number of remarkable distinct features characterize the plastome of *A. unedo*: five-fold dismissing of the SSC region in relation to most angiosperms; complete loss or pseudogenization of a number of essential genes; duplication of the *ndhH-D* operon and its location within the two IRs; presence of large tandem repeats located near highly re-arranged regions and pseudogenes. All these features outline the primary evolutionary split between Ericaceae and other ericalean families. The newly sequenced plastome of *A. unedo* with the available asterid sequences allowed the resolution of some uncertainties in previous phylogenies of Asteridae.

## Introduction

In vascular plants, the chloroplast genome (plastome) generally consists of a 120 to 160 Knt sized circular molecule of double stranded DNA whose gene content, gene order and genome organization are highly conserved [1]. In spite of their highly conserved nature, chloroplast genomes undergo recombination and rearrangements that result in deviations from the general rules. Completely sequenced chloroplast genomes provide valuable information about the duplication, loss, and transfer events in chloroplast genomes, and phylogenetic data to resolve relationships among major groups of plants such as angiosperms [2]. Moreover, the availability of an increasing number of complete chloroplast genome sequences can also be considered a major step forward towards exploiting the usefulness of chloroplast genetic engineering technology [3]. The immense technical progress in DNA sequencing has allowed for a dramatic increase in the number of completely sequenced chloroplast genomes in the last few years. Nowadays, nearly 250 plastomes from Streptophyta are available in the NCBI genome database, from which c.a. 95% correspond to vascular plants. Eudicots account for 130 completely sequenced plastid genomes, from which less than c.a. 30% correspond to Asteridae. This plant group encloses 102 families and 10 orders, being Cornales, Ericales, and Aquifoliales dated in the Early Cretaceous period the most ancient [4]. Family interrelationships are fully, or almost fully, resolved with medium to strong support except within the order Ericales [5], [6]. Ericales include 25 families, 346 genera, and 11,545 species. Currently Ericales contain c.a. 5.9% of eudicot diversity, of which one third is made up of Ericaceae alone [7]. Ericaceae, the heather family, is a large and diverse group of flowering plants composed of eight subfamilies (Enkianthoideae, Monotropoideae, Arbutoideae, Cassiopoideae, Ericoideae, Harrimanelloideae, Styphelloideae and Vaccinioideae) [8]. Arbutoideae is an understudied monophyletic group consisting of six genera: *Arbutus* L., *Arctostaphylos* Adans., *Arctous* Nied., *Comarostaphylis* Zucc., *Ornithostaphylos* Small., and *Xylococcus* Nutt. They are dry-adapted sclerophyllous taxa and most of the diversity in this group is in regions of Mediterranean climate in western North America [9]. Phylogenetic analyses within Arbutoideae suggested that *Arbutus* is not monophyletic [9]. The genus *Arbutus* includes approximately 11 species, four of them native to the Mediterranean region: *A. unedo*, *A. andrachne, A. pavarii* and *A. canariensis,* the last one being endemic to the Canary Islands. The remaining eight species of *Arbutus* occur in Western North America. *Arbutus unedo* L. (strawberry tree) is an evergreen shrub, or small tree, with a circum-Mediterranean range, growing in temperate regions where the highest temperatures occur simultaneously with the lowest rainfall [10].

At present, *Camellia sinensis* (Theaceae) (accession NC_020019), *Vaccinium macrocarpon* (Ericaceae) [11] and *Ardisia polysticta* (Primulaceae) [12] are the only three species of Ericales whose chloroplast genome has been completely sequenced. Here, we present the complete chloroplast genome sequence of *Arbutus unedo* using 454 Pyrosequencing technologies, thus contributing to increase the number of available complete sequence analyses of cpDNAs from Ericales. Comparative analyses will provide a valuable source of information about major restructuring events occurring during the evolution of ericalean chloroplast genomes, and phylogenetic data to resolve uncertain phylogenetic relationships within Asteridae. Moreover, the availability of the complete sequence of the chloroplast genome of *A. unedo* will be also highly valuable to subsequently exploit the usefulness of chloroplast genetic engineering, and to shed light on the molecular basis of the eco-physiological strategies which permit Mediterranean plants to thrive under very restrictive conditions.

## Materials and Methods

### Chloroplast Isolation and DNA Sequencing

Fresh material of *Arbutus unedo* L. was collected from a wild population at Montes de Toledo (N 39.49305 W 005.12211, Cáceres, Spain) and stored at −80°C. *A. unedo* is not considered a protected species and specific permissions were not required for collecting material in the specified location. However, it is noteworthy that *A. unedo* is a protected species in other localities in Spain (e.g. Madrid) and can be present within protected areas such as National Parks (these are not the cases for the plant material used in this study). The isolation of chloroplasts, and further DNA extraction and purification, were performed according to [13] with some modifications by Dr. J. Pérez in Secugen (http://www.secugen.es/). The purified DNA was sheared by nebulization, subjected to 454 library preparation and sequenced using Genome Sequencer (GS) FLX Titanium at Lifesequencing facilities (Parc Cièntific, Universitat de València, Spain).

### Genome Assembly and Annotation

The obtained nucleotide sequence reads were assembled using Mira assembly software [14]. The chloroplast genome reads were retrieved by comparison with the asterids chloroplast genomes downloaded from NCBI in a local BLAST database [15] and mapping all the reads with the complete chloroplast CDS set of *Panax ginseng* and *ycf15* gene from *Solanum lycopersicum*, this pre-assembly was used as our reference assembly (RA). The captured reads were de novo assembled with the “uniform read distribution (-urd)” option as this allows repeats to be disentangled during the contig building phase, maintaining the average coverage multiplied by a value of 1.5, separating IR zones and repeats. Then, we mapped the rest of the reads to the RA with the “also build new contigs (-abnc)” option, making new contigs with reads that did not map to the backbone. Finally, contigs were filtered and ordered by aligning them to the RA using the BLAST program, and jointed with gap4 from the Staden package [16]. Gap regions, IR-LSC and IR-SSC junctions were PCR amplified with LA Taq (Takara Bio Inc., Shiga, Japan) with specific primers (Table S1) on a 96-well SensoQuest labcycler, PCR products were visualized on 2% agarose gels. DNA was purified using Illustra GFX PCR DNA and Gel band Purification kit (GE Heathlcare Life Science, Buckinghamshire, England) and sequenced with an ABI 3100 Genetic analyzer using the ABI BigDyeTM Terminator Cycle Sequencing Ready Reaction Kit (Applied Biosystems, Foster City, California). Long fragments had to be cloned using the TOPO XL cloning kit (Invitrogen, Carls-bad, CA) and sequenced by “primer walking”. In all cases, samples were sequenced in both forward and reverse directions. Open reading frames (ORFs) were identified using Artemis [17] and functional assignments were made based on the sequence similarity of BLASTp, BLASTx and BLASTn searches against NCBI databases. Transfer and ribosomal RNA genes were identified using tRNAscan-SE [18], Rfam [19] and RNAweasel [20]. All delimited genes were carefully revised in order to assess correct reading frames and intron limits in the case of protein-encoding genes. Thereby, we compared all reading frames with other angiosperms considering the possible creation of start and stop codons by editing in some cases and searched for sequence motif characteristics at both 5′ and 3′ ends of group II introns in the case of intron-bearing genes. Delimitation of rRNAs was made on the basis of their structural features with the aid of Mfold [21]. The graphical map of the circular plastome of *A. unedo* was drawn with Organellar Genome DRAW (OGDRAW) [22]. For general manipulations of sequences we used Geneious [23] and CLC Sequence Viewer available at http://www.clcbio.com/products/clc-sequence-viewer/(this last program was also used in the construction of genetic maps). The obtained nucleotide sequence is available at the GenBank sequence database provided by the National Center for Biotechnology Information (NCBI) with the accession number JQ067650.

### Phylogenetic Analyses

Phylogenetic reconstructions were performed on the basis of 83 chloroplast genes from 57 species (see Table S2 for accession). Alignments were performed with Muscle [24] and trimmed with GBLOCKs [25] with default parameters. The sequences matrix for each gene was subjected to JModelTest to find the best-fit evolutionary model [26]. In order to test the phylogenetic signal TREE-PUZZLE was used [27]. For maximum-likelihood (ML) analyses, the concatenated nucleotide matrix of 57 taxa, and 55016 nt was analyzed with RAxML v. 7.2.8 [28] using the GTRGAMMA and a bootstrap analysis with 500 replicates. The Bayesian analyses were implemented with Mr Bayes V.2.1.0 [29]. The concatenated nucleotide matrix was analyzed using: GRT +I+ G model (4 discrete rate categories by default). Markov chain Monte Carlo (MCMC) analyses were run for 5,000,000 generations, and four independent Markov chains. Trees and model parameters: trees were sampled every 1000 generations. Stationarity was assessed by examining the standard deviation of split frequencies and by plotting the –ln Likelihood per generation using Tracer v1.4 [30], and trees generated before stationarity were discarded. The majority rule consensus tree produced by MrBayes was drawn with FigTree [31].

### Additional Analyses

Whole genome alignments were performed with MultiPipMaker [32]. Gene map and alignments of the LSC region were performed with MAUVE [33] implemented in Geneious [23]. The frequency of codon usage was deduced on the basis of the sequences of protein-coding genes within the cpDNA with the assistance of the program DnaSP, version 5.1. [34]. Tandem within the cpDNA of *A. unedo* and other asterids were found by using the program “Tandem repeats finder” [35].

## Results and Discussion

### Genome Organization and Gene Content of the *A. unedo* Plastome

**Figure 1:**
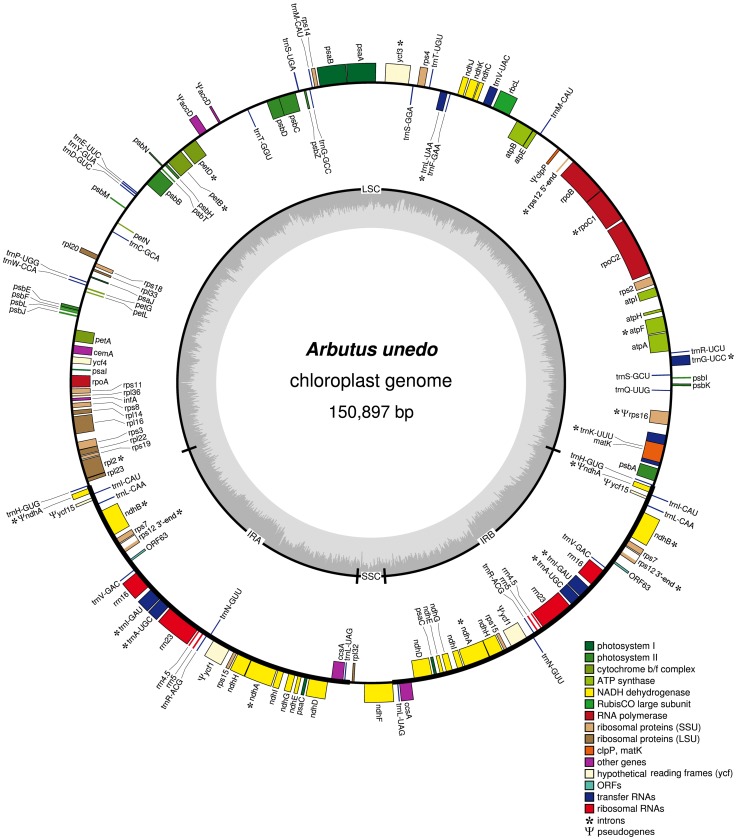
Gene map of the *Arbutus unedo* complete chloroplast genome represented as a circular molecule. Genes shown inside the circle are transcribed clockwise and genes outside are transcribed counter clockwise. Genes for tRNAs are represented by one letter code amino acids with anticodons. Asterisks indicate genes with introns. Pseudogenes are preceded by the Ψ symbol.

The chloroplast genome of *Arbutus unedo* (Figure 1) is a circular molecule of 150,897 nt within range of other angiosperms. The cpDNA of *A. unedo* is structured in the typical quadripartite structure, consisting of two inverted repeats (IRa and IRb) separated by large single copy (LSC) and small single copy (SSC) regions (Figure 1). The GC content of the *A. unedo* cpDNA is 37.31%, similar to the other reported cpDNA genomes from asterids. The GC content of the LSC and SSC are 35.64% and 28.94%, respectively, whereas that of the IR regions is 40.55%. The *A. unedo* cpDNA contains a total of 142 genes from which 114 have a single copy, whereas 28 are duplicated (Table 1). Two copies of each of the four genes encoding the chloroplast rRNAs (*rrn23*, *rrn16*, *rrn5* and *rrn4.5*) are distributed throughout the IRs. The tRNAs are encoded by twenty-one single-copy and nine two-copy genes distributed throughout the LSC region and the IRs, respectively. There are 87 genes encoding putative functional proteins. Twelve full-length and functional protein-encoding genes have two copies located in the IRs. Thirty-eight genes encode proteins related to photosynthesis: 8 for the photosystem I, one of them (*psaC*) in two copies; 15 for the photosystem II; 6 for the cytochrome b6/f complex; 6 for the ATP synthase; one for the Calvin Cycle; and two copies of the *ccsA* for the synthesis of C-type cytochrome. Thirty genes encode proteins related with the gene expression machinery involved in transcription, splicing and translation: 4 for the RNA polymerase; 9 for the ribosomal large subunit; 15 for the ribosomal small subunit, three of them (*rps7*, *12* and *15*) in two copies; one for maturase K; and one for the translation initiation factor 1. Eighteen genes encode proteins for the NADH-dehydrogenase complex involved in chlororespiration: three of them were located within the LSC region (*ndhC*, *K* and *J*), one was found within the SSC region (*ndhF*) and seven were located within the IRs (*ndhA*, *B*, *D*, *E*, *G*, *H*, and *I*) each of them in two copies. Finally, the *cemA* gene encoded for an envelope membrane protein. In the cpDNA of *A. unedo* there are 15 different genes harbouring introns (note that some of them are duplicated, see below), which are cis-spliced (Table 2). Fourteen genes have a single intron (8 protein-coding and 6 tRNA-coding genes), whereas a single gene (*ycf3*) contains two introns. Out of the 16 genes with introns, 12 are located in the LSC (8 protein-coding and 4 tRNA genes), 4 are located in two copies in each of the IRs (2 protein-coding and 2 tRNA genes). The *trnK-*UUU gene has the largest intron (2,559 nt) and contains an ORF encoding the *matK* gene. This gene encodes a maturase that preferentially catalyses splicing of the *trnK* intron, but it may also have a generalist function.

**Table 1:** Genes found in the *Arbutus unedo* chloroplast genome.

Function	Different products	Total genes	Total introns	Gene name
Photosystem I	7	8	0	*psaA, B, C* c, *I, J, ycf3* a, *ycf4*
Photosystem II	15	15	0	*psbA, B, C, D, E, F, H, I, J, K, L, M, N, T, Z*
Cytochrome b6/f complex	6	6	2	*petA, B* b, *D* b, *G, L, N*
ATP synthase	6	6	0	*atpA, B, E, F* b, *H, I*
Calvin clycle	1	1	0	*rbcL*
C-type cytochrome synthesis	1	2	0	*ccsA* c
NADH dehydrogenase	11	18	4	*ndhA* ^bc^, *B* ^bc^, *C, D* c, *E* c, *F, G* c, *H* c, *I* c, *J, K*
RNA polymerase	4	4	0	*rpoA*, *B*, *C1*, *C2*
Maturase K	1	1	0	*matK*
Translation initiation factor	1	1	0	*infA*
Large subunit ribosomal proteins	9	9	1	rpl2 b, 14, 16, 20, 22, 23, 32, 33, 36
Small subunit ribosomal proteins	12	15	3	rps2, 3, 4, 7^c^, 8, 11, 12^cd^, 14, 15^c^, 18, 19
Ribosomal RNAs (4)	4	8	0	rrn23^c^, rrn16^c^, rrn5^c^, rrn4.5^c^
tRNAs	30	39	8	trnA-UGC^bc^, C-GCA, D-GUC, E-UUC, F-GAA, G-GCC, G-UCC^b^, H-GUG^c^, I-CAU^c^, I-GAU^bc^, K-UUU^b^, L-CAA^c^, L-UAA^b^, L-UAG^c^, M-CAU, fM-CAU, N-GUU^c^, P-UGG, Q-UUG, R-ACG^c^, R-UCU, S-GCU, S-GGA, S-UGA, T-GGU, T-UGU, V-GAC^c^, V-UAC^b^, W-CCA, Y-GUA
Envelope membrane protein	1	1	0	*cemA*
Pseudogenes	5	8	0	*accD, clpP, ndhA* c, *rps16* b, *ycf1* c, *ycf15* c

a Gene containing two introns.

b Gene containing a single intron.

c Two gene copies in the IRs.

d Gene whose transcripts are trans-spliced.

**Table 2:** Genes having cis-spliced introns in the *Arbutus unedo* cpDNA and the lengths of exons and introns.

Gene	Location	Exon I nt	Exon II nt	Exon III nt	Intron I nt	Intron class	Intron II nt	Intron class
*atpF*	LSC	145	410	–	714	IIA	–	–
*ndhA*	IR	553	539	–	1073	IIB	–	–
*ndhB*	IR	777	756	–	684	IIB	–	–
*petB*	LSC	6	642	–	736	IIB	–	–
*petD*	LSC	8	481	–	792	IIB	–	–
*rpl2*	LSC	391	434	–	672	IIA	–	–
*rpl16*	LSC	9	408	–	10367	IIB	–	–
*rpoC1*	LSC	453	1626	–	738	IIB	–	–
*rps16*	LSC	40	188	–	857	IIB	–	–
*trnA*-UGC	IR	37	35	–	807	IIA	–	–
*trnG*-UCC	LSC	23	48	–	692	IIB	–	–
*trnI*-GAU	IR	37	35	–	950	IIA	–	–
*trnK*-UUU	LSC	37	35	–	2514	IIA	–	–
*trnL*-UAA	LSC	35	50	–	521	I	–	–
*trnV*-UAC	LSC	39	35	–	620	IIA	–	–
*ycf3*	LSC	124	230	153	680	IIB	722	IIB

The cpDNA of *A. unedo* contains a lower number of codons (17,980) in comparison to other angiosperms [e.g. *Ageratina adenophora* with 24,894 and *Vigna radiata* with 26,274 (Table S2)]. This is possibly due to the pseudogenization of numerous and large ORFs in the *A. unedo* chloroplast genome, and the loss of the *ycf2* gene, since the cpDNAs of the three plant species are very similar in size (150,698 nt for *A. adenophora*, 151,271 for *V. radiata* and 150,897 for *A. unedo*). Table 3 show the frequency of codon usage deduced on the basis of the sequences of protein-coding genes. Leucine was seen to be the most frequent amino acid, with 759 codons encoding this amino acid (10.7%), while cysteine was the least frequent, with 43 codons (1.13%). The codon usage in *A. unedo* was biased toward high representation of A and T at the third codon position (72.4%), similar to the cpDNA from other Angiosperms [e.g. *Ageratina adenophora* and *Vigna radiata* (Table S2)].

**Table 3:** Codon-anticodon recognition pattern and codon usage for the chloroplast genome of *Arbutus unedo*.

Amino acid	tRNA	Codon	No.*	Amino acid	tRNA	Codon	No.*	Amino acid	tRNA	Codon	No.*
Ala	trnA-UGC	GCU	497	Lys	trnK-UUU	AAA	678	Ser	trnS-GCU	AGU	254
	trnA-UGC	GCA	305		trnK-UUU	AAG	191		trnS-GCU	AGC	69
	trnA-UGC	GCC	172	Leu	trnL-CAA	UUG	380		trnS-GGA	UCU	402
	trnA-UGC	GCG	130		trnL-UAA	UUA	685		trnS-GGA	UCC	197
Cys	trnC-GCA	UGU	160		trnL-UAG	CUU	405		trnS-UGA	UCA	232
	trnC-GCA	UGC	43		trnL-UAG	CUA	242		trnS-UGA	UCG	96
Asp	trnD-GUC	GAU	521		trnL-UAG	CUC	116	Thr	trnT-GGU	ACU	407
	trnD-GUC	GAC	134		trnL-UAG	CUG	97		trnT-GGU	ACC	176
Glu	trnE-UUC	GAA	670	Met	trnM-CAU	AUG	441		trnT-UGU	ACA	286
	trnE-UUC	GAG	220	Asn	trnN-GUU	AAU	591		trnT-UGU	ACG	91
Phe	trnF-GAA	UUU	676		trnN-GUU	AAC	168	Val	trnV-GAC	GUU	385
	trnF-GAA	UUC	311	Pro	trnP-UGG	CCU-P	295		trnV-GAC	GUC	133
Gly	trnG-GCC	GGU	445		trnP-UGG	CCA-P	223		trnV-UAC	GUA	392
	trnG-GCC	GGC	153		trnP-UGG	CCC-P	145		trnV-UAC	GUG	137
	trnG-UCC	GGA	549		trnP-UGG	CCG-P	95	Trp	trnW-CCA	UGG	317
	trnG-UCC	GGG	220	Gln	trnQ-UUG	CAA	497	Tyr	trnY-GUA	UAU	542
His	trnH-GUG	CAU	341		trnQ-UUG	CAG	136		trnY-GUA	UAC	117
	trnH-GUG	CAC	87	Arg	trnR-ACG	CGA	282	Stop	–	UAA	38
Ile	trnI-CAU	AUA	490		trnR-ACG	CGU	280		–	UAG	17
	trnI-GAU	AUU	759		trnR-ACG	CGG	65		–	UGA	18
	trnI-GAU	AUC	292		trnR-ACG	CGC	62				
					trnR-UCU	AGA	306				
					trnR-UCU	AGG	86				

* Numerals indicate the frequency of usage of each codon in 17,947 codons in 73 potential protein-coding genes.

### Major Restructuring of the *A. unedo* Plastome

**Figure 2:**
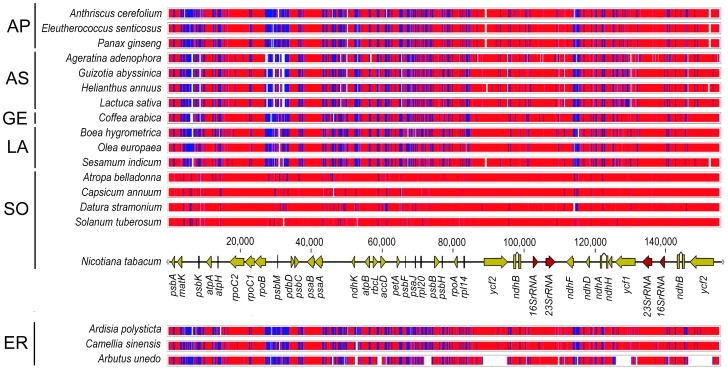
Whole genome alignment of the *Arbutus unedo* chloroplast genome with other asterid chloroplast genomes obtained with MultiPipMaker [32] taking that of *Nicotiana tabacum* as the reference. Sequence identity is shown by red (75–100%), green (50–75%), and white (<50%). Positions of some genes in *N. tabacum* are indicated as a guide (genes encoding proteins and rRNAs are indicated as yellow and red arrows, respectively). The taxonomic classification is indicated on the left (AP: Apiales, AS: Asterales, GE: Gentianales, LA: Lamiales, SO: Solanales, ER: Ericales).

**Figure 3:**
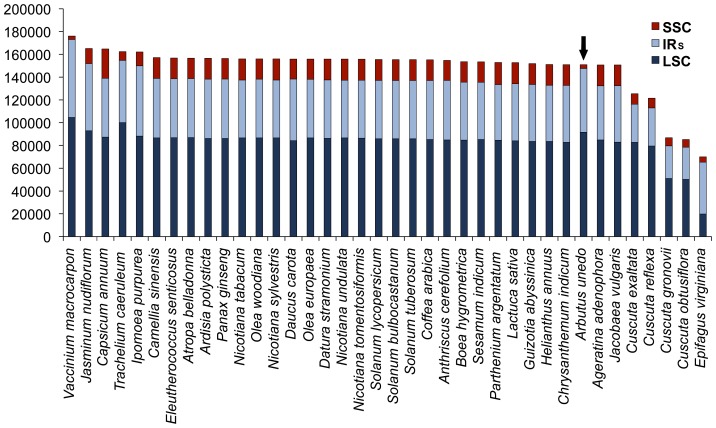
Comparison of the lengths of LSC, SSC and IR regions among Asteridae. Accession numbers of the corresponding genomes are indicated in Table S2.

**Figure 4:**
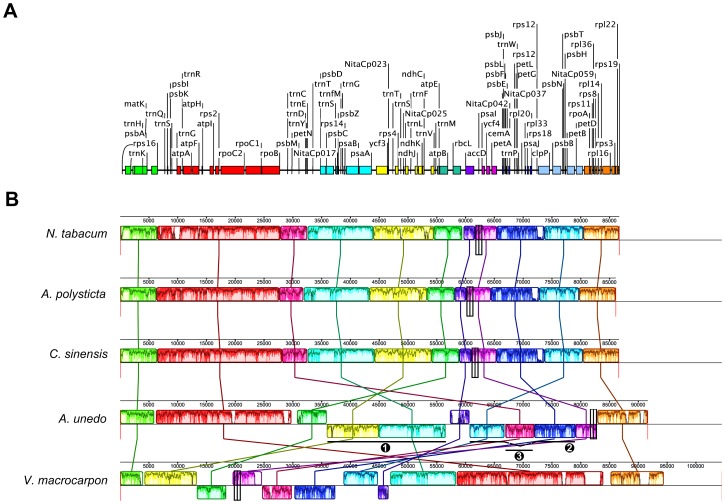
Gene map and alignment of the LSC region of three ericalean species in relation to *Nicotiana tabacum*. (A) Gene map of the LSC region in the chloroplast genome of *Nicotiana tabacum*. (B) Gene alignment of the LSC region of *Ardisia polysticta*, *Camellia sinensis*, *Arbutus unedo*, *Vaccinium macrocarpon* belonging to Ericales and *Nicotiana tabacum* belonging to Solanales. MAUVE multiple alignment [33] implemented in Geneious [23]. Colored outlined blocks surround regions of the genome sequence that aligned with part of another genome. The coloured bars inside the blocks are related to the level of sequence similarities. Lines link blocks with homology between two genomes. Accession numbers of the corresponding genomes are indicated in Table S2.

The whole-genome alignment of the *A. unedo* cpDNA with other Asteridae (Figure 2) showed high conservation of many coding regions along with remarkable rearrangements. The gene order was compared taking *N. tabacum* as a reference since *Nicotiana* is considered to have the ancestral angiosperm gene order [36]. As shown in Figure 2, the cpDNA of *A. unedo* clearly deviates from that of *N. tabacum* to a greater extent than other asterids because of extensive rearrangements. The higher divergence is observed in a portion comprised within position 90,000 and the end of the sequence, which includes the two IRs and the SSC region. Comparisons of the lengths of the three different regions of the plastome within asterids (Figure 3) revealed a remarkable shortness of the SSC region in comparison with most of asterids, and also most of the other angiosperms with an average size of c.a. 18,000 nt. This feature was exclusively found in the two Ericaceae whose cpDNA has been sequenced to date: *Arbutus unedo* (3,400 nt, this study) and *Vacccinium macrocarpon* (3,029 nt, Table S2). The reduction of the SSC region in these two Ericaceae was even higher than in non-photosynthetic parasitic plants such as those belonging to the genera *Cuscuta* and *Epifagus*, which have extraordinary reduction of their entire chloroplast genomes (Figure 3). The extreme shortening of the SSC regions results from the duplication and inclusion of the entire *ndhH-D* operon within each of the two IR regions which are extended to 34,232 nt in *V. macrocarpon* but not *A. unedo*. The conservation of the regular sizes of the IRs in *A. unedo* was mainly due to the loss of the *ycf2* gene consisting of c.a. 7,000 nt that partially compensates the gain of the *ndhH-D* operon (Figure 1). Figure S1 shows different gene arrangements found in the SSC region including two algae belonging to two different phyla (Streptophyta and Chlorophyta). The most frequent gene arrangement is represented by *Nicotiana tabacum* (Figure S1B), which was present in c.a. 75% of angiosperms whose cpDNA has been completely sequenced. The arrangement shown in algae such as *Chara vulgaris* (Figure S1A) and *Nephroselmis olivacea* (Figure S1C) belonging to Streptophyta and Chlorophyta, respectively, show remarkable similarities to those of vascular plants. Similar to the SSC region, the gene content in the two IRs is rather well conserved among plants. Figure S2 shows different gene arrangements found in the IRs including the alga *Chara vulgaris*. Almost 70% of Asteridae whose chloroplast genome has been completely sequenced had the general gene content and order of *N. tabacum* (Figure S2E). Similarly to the SSC and IRs, the LSC show several relocations of genes in the *A. unedo* plastome. Figure 4 shows preserved co-localization of genes on chromosomes of different species (shared or conserved synteny) within the LSC regions of the cpDNAs of four ericalean species (*Ardisia polysticta*, *Camellia sinensis*, *Arbutus unedo* and *Vaccinium macrocarpon*) and *Nicotiana tabacum*. *A. polysticta*, *C. sinensis* and *N. tabacum* exhibit a conserved synteny, whereas *A. unedo* and *V. macrocarpon* show extensive rearrangements resulting in a considerable loss of synteny. We hypothesize that the LSC region of the *A. unedo* plastome had experienced at least two main inversions of segments. One of them [(1) in Figure 4] could include the segment between *trnT*-GUU and *trnV*-UAC. The other one [(2) in Figure 4] could include the segment between *psaI* and *petD*. A minor additional inversion could involve a segment between *trnC*-GCA and *trnE*-UUC and comprising of the *petN* and *psbM* genes [(3) in Figure 4], which is inserted within the segment (2). The complex pseudogenization process that occurred on *Arbutus* LSC (see below) which affects both the *accD* and *clpP* genes may be a clue to support our hypothesis about these inversion endpoints. The most parsimonious interpretation of the distribution of the cpDNA inversions outlines a primary evolutionary split between Ericaceae and Theaceae.

### Losses and Pseudogenization of Essential Genes

The number of genes and their order are generally conserved in the chloroplast genomes of most angiosperms. However, as the availability of sequenced genomes has increased, a number of exceptional gene losses have been identified (summarized in [37]). The *rpl33* gene is lost in *Phaseolus vulgaris* and *Vigna radiata*; the *infA* gene is lost in almost all rosid species; the *rpl32* gene is lost in the *Populus* genus; the *rps16* is lost in *Medicago truncatula*, *Phaseolus vulgaris*, *Cicer arietinum*, *Vigna radiata* and the *Populus* genus; the *ycf1*, *ycf2* and *accD* genes in Poaceae (Table S2). Many gene losses have been interpreted as transfers to the nucleus. After analysing the gene content of the cpDNA of *A. unedo*, we found several genes which appeared either lost, such as *ycf2,* or non-functional, such as *clpP1*, *accD*, *ycf1* and *ycf15* (Figure 1).

The chloroplast genome of most plants and several algae contains two large open reading frames known as *ycf1* and *ycf2* encoding proteins of 1901 and 2280 amino acids in tobacco, which are essential for cell survival [38]. In most land plants, two identical *ycf2* copies are located in the IR regions. However, independent losses of the *ycf2* gene occurred in various angiosperms [39]. In *A. unedo* the *ycf2* gene is completely absent, whereas the *ycf1* gene remains residual as a pseudogen in two copies within each IR region (Figure 1 and Figure S2C). After reviewing the status of these two genes among asterids, we found that the *ycf2* gene was only completely lost in the cpDNA of *A. unedo*. We also found non-functional forms of this gene in other Asteridae (e.g. *V. macrocarpon* and *T. caeruleum* (Table S2). The functionality of some other *ycfs,* apart from *ycf1*, *ycf2*, *ycf3* and *ycf4,* has been questioned by their relatively frequency as pseudogenes. This is the case of *ycf15* found as pseudogen in *A. unedo* and also in other asterids. The *ClpP1* gene encodes a caseinolytic protease which has been found in almost all bacterial species and eukaryotic organelles [40]. This gene is present in all plant lineages with a few exceptions being essential for plant development in tobacco [41]. In this study, we found that the *clpP* gene appears as a non-functional pseudogene exclusively in the two analysed Ericaceae (*A. unedo* and *V. macrocarpon*). In this study, we found the presence of the *accD* gene as residual pseudogene in the cpDNA of three asterids: *A. unedo, V. macrocarpon* and *T. caeruleum*. This gene encodes one of the four subunits that constitute the plastid Acetyl-CoA carboxilase (ACCase) which catalyzes the formation of malonyl-CoA in fatty acid synthesis. The *rps16* gene for ribosomal protein S16 (*rps16*) which is generally encoded in the chloroplast genome of flowering plants, is interrupted by two stop codons in *A. unedo*. This gene appears non-functional in several plant lineages and is replaced by nuclear genes [42].

The essentiality of the *ycf1*, *ycf2*, *clpP*, *accD* and *rps16* genes and their absence or presence as pseudogenes suggested that they could be substituted by nuclear-encoded versions. Hence, we hypothesize the possible transference of copies of these essential genes to the nucleus. Further studies based on searches of nuclear-encoded copies of these genes along with verification of their expression, targeting to the chloroplast and its correct functioning will be necessary to test this hypothesis. From a practical point of view, extensive rearrangements and pseudogenizations may have consequences when designing appropriate transformation vectors to express transgenes. To date, at least 14 different insertion sites were proposed for the targeting of transgenes within the chloroplast genome [43]. A number of these sites are inapplicable due to pseudogenizations and rearrangements in the cpDNA of *A. unedo* (e.g. *rbcL/accD*, *5′rps12/clpP*, *petD/rpoA*). This fact stresses the importance of having the complete sequence of the chloroplast genome of a plant species in order to design a successful protocol of transformation.

### Large Tandem Repeats are Found in the *A. unedo* Plastome

**Figure 5:**
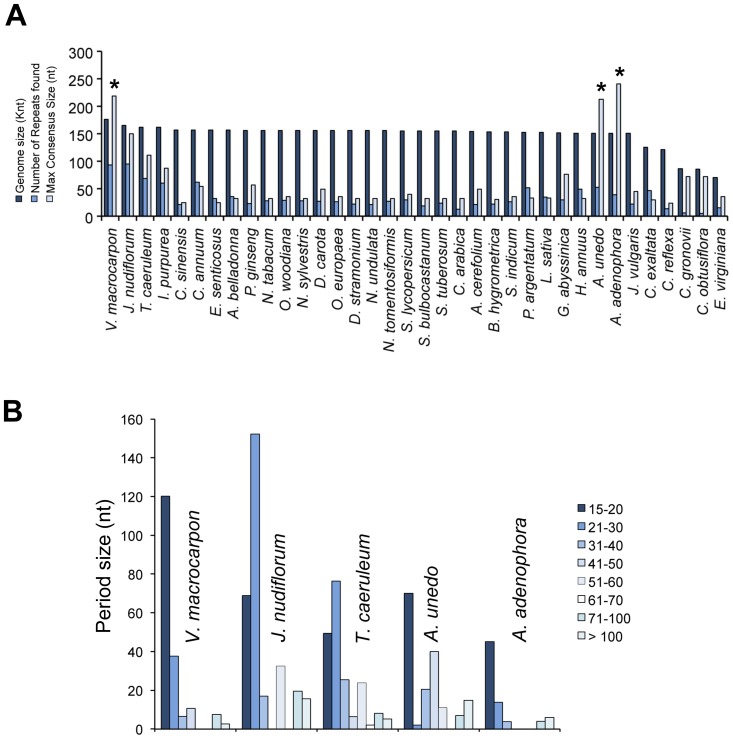
Tandem repeats in the *Arbutus unedo* plastome and other asterids. (A) Genome sizes, number of repeat found and maximum consensus size of some asterids arranged by their genome size. (B) Frequency of tandem repeats by length.

Tandem repeats (TRs) are ubiquitous, unstable genomic elements, which have historically been designated as non-functional DNA. However, mutations in these repeats often have notorious phenotypic consequences. Some of these mutations are deleterious such as those causing diseases in humans, whereas others are beneficial such as those conferring useful phenotypic variability [44]. In yeasts and humans, TRs are frequently found in promoters and are directly responsible for the divergence in transcription rates [45]. In this study we searched for tandem repeats within the cpDNA of *A. unedo* and other asterids by using the program “Tandem repeats finder” [35]. A total of 53 TRs were found in *A. unedo*. This number was only surpassed by five asterid species out of the 36 studied (Figure 5). The remaining species had an average of 30 TRs (except non-photosynthetic parasitic plants whose cpDNA is highly reduced). Generally, the species with higher number of TRs also show the largest genome sizes (Figure 5). However *A. unedo* was an exception. This species had one of the smallest genome sizes among the analysed asterid species, but it had one of the highest numbers of TRs (Figure 5).

Recently, a new class of large TR has been discovered in the pathogenic yeast *Candida glabrata*, which are termed megasatellites. These TRs are DNA tandem arrays made of large motifs widespread in this species (40 copies in a genome of 12.34 Mb), which seem to promote genome rearrangements by interfering with DNA replication (reviewed in [46]). In our analysis, we found TRs of more than 150 nt of period size (megasatellites) in only four Asteridae: *V. macrocarpon* (219 nt), *A. unedo* (213 nt), *A. adenophora* (241 nt) and *J. nudiflorum* (150 nt), whereas most of the studied species showed consensus sizes smaller than 50 nt. In general, species with a high number and/or large amount tandem repeats (more than 52 tandem repeats and/or 100 nt of consensus size) showed extensive rearrangements and/or pseudogenizations. Interestingly, in *A. unedo* the larger TRs (213 and 117 nt) were found near the *clpP* and *accD* pseudogenes. Smaller TRs were also found near the two copies of the *ycf1* pseudogen. More exhaustive studies would be necessary to establish relationships between the presence of certain TRs and genome rearrangements, pseudogenizations and/or transference of genes from chloroplasts to the nucleus.

### Seven Out of 11 Plastid-encoded *ndh* Genes are Duplicated in *A. unedo*

The chloroplast NAD(P)H dehydrogenase (Ndh) complex is involved in photosystem I (PSI) cyclic electron transport and chlororespiration (reviewed in [47]). Several studies have suggested that the chloroplast NDH complex is involved in protective or adaptive mechanisms of plants to different stresses, which increase reactive oxygen species (ROS) formation and cause oxidative stress e.g. [48]–[50]. The chloroplast Ndh complex includes 11 subunits encoded by the chlororoplast *ndh* genes, which are widespread among the three regions of the plastome of most plants. Six *ndh* genes constitute the *ndhH-D* operon located within the SSC region in most plants. The genes of this operon are co-transcribed forming a 7–8 Kb primary transcript, which undergo a series of posttranscriptional processes including intercistronic cleavages, intron splicing and C to U editing. Such posttranscriptional modifications have consequences on gene expression modulating differential transcript levels and thereby the corresponding proteins (e.g. [51]–[54]). The two Ericaceae *A. unedo* presented here and *V. macrocarpon* [11] are the only two species which show a duplication of the entire *ndhH-D* operon among all streptophytes whose cpDNA has been sequenced to date. In other plants, only partial duplications of the operon can be found [e.g. *Trachelium caeruleum* and *Ipomoea purpurea* among Asteridae; *Pelargonium x hortorum* and *Monsonia speciosa* among Geraniaceae (Figure S3 and Table S2)]. It is noteworthy that generally cpDNAs with unusually duplicated *ndh* genes exhibit extensive rearrangements and a higher frequency of pseudogenes. The possible causal link among these three features remains to be determined. Repeated duplication of some chloroplast-encoded genes such as the *clpP* correlated with an increase of synonymous substitution rates and positive selection of the resulting protein in certain plant lineages [55]. In order to test if this was a more general rule extendible to the *ndh* genes, we obtained an estimate of the synonymous substitution rates by using the program DnaSP 5.1 [34]. As shown in Figure S4, we found a low ω, dN/DS or Ka/Ks ratio (ratio of the number of non-synonymous substitutions per non-synonymous site) in all cases (<<1). This means that the studied proteins seem to undergo purifying selection instead of positive selection. Surprisingly, this conservationism is also found in *V. macrocarpon*, which shows pseudogenization of three *ndh* genes (*ndhG*, *ndhI* and *ndhK*).

All the contrasting features regarding the *ndh* genes found in *A. unedo* and *V. macrocarpon*, makes these two plant species exceptionally interesting when investigating the functionality of the chloroplast NDH complex at different levels such as gene expression and its regulation; stoichiometry among NDH subunits; structure of the NDH complex and its interactions with other(s) thylakoid complex(es); enzymatic properties, etc. From an ecophysiological perspective, there is consensus in that chlororespiration and the NDH complex are not relevant under non stressful conditions but, they should be indispensable to prevent the over-reduction of intermediates of the photosynthetic electron transport and the concomitant ROS production under stress [56]. The difference in relation to the *ndh* genes found in *A. unedo* and *V. macrocarpon* with respect to other plans and between them open new perspectives to test the involvement of NDH complex, and possibly other components of the chlororespiratory pathway in the adaptation of Mediterranean plants to highly fluctuating and often stressful environmental conditions.

### Application of Parallel Sequencing of Chloroplast Genomes to Resolve Phylogenetic Relationships within Asterids

**Figure 6:**
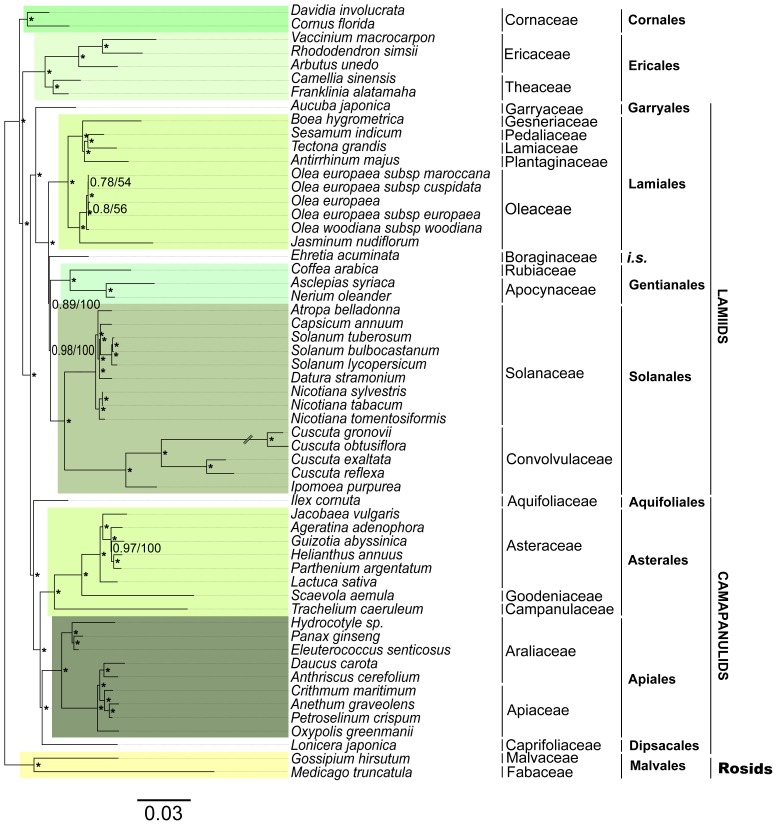
Phylogram based on sequence analysis of 83 chloroplast genes from 57 plant species (Table S2). Asterisks indicate nodes with values of 0.1 and 100 for bootstrap values and posterior probabilities, respectively. The scale bar indicates substitutions/site. The current taxonomic classifications are indicated on the right (*i*.*s*., *incertae sedis*).

The Asteridae represent an evolutionary successful group with over 80,000 species or 1/4–1/3 of all flowering plants. The phylogeny of asterids has been explored with analysis of a number of chloroplast-encoded genes resolving with strong support basal interrelationships among Cornales, Ericales, Lamiidae, and Campanulidae [4]. However, the relative positioning of the orders Gentianales, Lamiales and Solanales within lamiids remains unresolved. In some cases, Solanales and Lamiales are grouped within the same clade, which does not include the order Gentianales [57] whereas in other cases, Gentianales and Lamiales are grouped within the same clade, which does not include Solanales [12], [58]. Here we present an updated phylogeny of Asteridae including 55 specimens from ten different orders (including five ericalean species) and two rosid species as outgroup (see Table S2 for accessions). All analyses were based on a nucleotide sequence alignment comprising 55016 nt including 83 chloroplast genes obtaining identical topologies after ML and Bayesian analyses. Figure 6 shows a phylogram whose topology is overall consistent with those of previously published phylogenetic reconstructions (e.g. [2], [12], [57], [58]). However, only in our phylogeny, and that of [2] Gentianales and Solanales are grouped within the same clade, which does not includes Lamiales. If we focus on the support of each clade in the different phylogenies, we obtained the highest values to date (0.98/100 for PP/BT). These results dissipate the uncertainty of the relationships among Gentianales, Lamiales and Solanales: Solanales and Gentianales seem to be more closely related to each other than to Lamiales. Probably, the support of the relationships among problematic taxa may be improved by increasing the number of species representatives of each taxa and the number of analysed sequences, as is the case of the three orders referred to above.

For future investigation, we propose sequencing the same 83 chloroplast genes and using a higher number of species representing each Ericalean family to resolve the uncertainty of interfamilial relationships within Ericales. This stresses the importance of sequencing more chloroplast genomes within this order. In this line, the generated gene sequences in this study alongside other available in Genbank, will be helpful for developing universal primers to further reveal the molecular phylogeny of Ericales, even at lower taxonomic levels including populations by sequencing more variable intergenic regions.

### Conclusions and Perspectives

*A. unedo* is the first Arbutoideae, second Ericaceae and third ericalean species whose plastome has been completely sequenced (January 2013), which shows a number of unusual features that can be further exploited in a variety of fields. Comparative studies of plastome architecture and tandem repeats would be a valuable source of information about the duplication, loss, and transfer events of chloroplast genes providing information about patterns of evolution. The complete loss or pseudogenization of a number of essential genes (*accD*, *clpP*, *rps16*, *ycf1*, *ycf2*) could allow studies about the putative presence of the corresponding nuclear-encoded genes, patterns of expression, structural features of the proteins, their import into the chloroplasts and possible physiological consequences. The duplication of the *ndhH-D* operon provides an extra-copy of each gene within the operon with respect to most plants and perhaps a “natural overexpression”. This particularity makes this plant species very interesting for the study of the expression and the physiological role of the chloroplast Ndh complex in relation to other plants with a single copy of the referred operon. Knowledge of the general structure and sequence of the *A. unedo* plastome, as well as gene losses or pseudogenizations and gene duplications, may be useful to study possible alterations in posttranscriptional events in relation to other well-studied plants, as well as being useful for exploiting chloroplast genetic engineering technology. Finally, in this study we show an improved phylogeny of asterids including 57 different species with a number of Ericales which resolves some uncertainties of previous phylogenies.

## Supporting Information

Figure S1 **Gene maps representative of the most recurrent variants of the SSC region in plants.** Accession numbers of the corresponding genomes are indicated in Table S2.
(TIF)

Figure S2 **Gene maps representative of the most recurrent variants of the IRs in plants.** Accession numbers of the corresponding genomes are indicated in Table S2.
(TIF)

Figure S3 **Gene map of the** ***ndhH-D*** **operon in plants showing examples of complete and partial duplications.** Coding regions are indicated as arrows. Duplicated portions are indicated in red. Introns and intergenic regions are indicated as thick and thin black bars, respectively. Accession numbers of the corresponding genomes are indicated in Table S2. The scale bar indicates positions in nt.
(TIF)

Figure S4 **dS and dN values of 17 chloroplast genes.** These genes are: *ndhA* 1017 nt; *ndhB* 1473 nt; *ndhC* 342 nt; *ndhD* 1306 nt; *ndhE* 303 nt; *ndhF* 2247 nt; *ndhG* 396 nt; *ndhH* 1179 nt; *ndhI* 487 nt; *ndhJ* 474 nt; *ndhK*; 675 nt; *rbcL* 1425 nt; *atpA* 1494 nt; *psbA* 957 nt; *cemA* 682 nt; *petA* 963 nt; *psbB* 1515 nt. Diagram shows the pairwise dS values and dN values along with the dN/dS values between six asterid species and the outgroup (*Gossypium hirsutum*), three Ericaceae (Au: *Arbutus unedo*; Rs: *Rhododendron simsii*; Vm: *Vaccinium macrocarpon*) and three Asteraceae (Aa: *Ageratina adenophora*; Ga: *Guizotia abyssinica*; Ha: *Helianthus annuus*). Accession numbers of the corresponding genomes are indicated in Table S2.
(TIF)

Table S1 **List of primers used to complete gap regions in IR-LSC and IR-SSC junctions.**
(DOCX)

Table S2 **List of taxa included in either text or figures with GenBank accession numbers and the corresponding bibliographic references.**
(DOCX)
