# Supplementary figures and images for: Balanced Gene Losses, Duplications and Intensive Rearrangements Led to an Unusual Regularly Sized Genome in Arbutus unedo Chloroplasts
Source: PLoS One. 2013 Nov 18;8(11):e79685. doi: 10.1371/journal.pone.0079685 (PMC3832540; doi:10.1371/journal.pone.0079685)

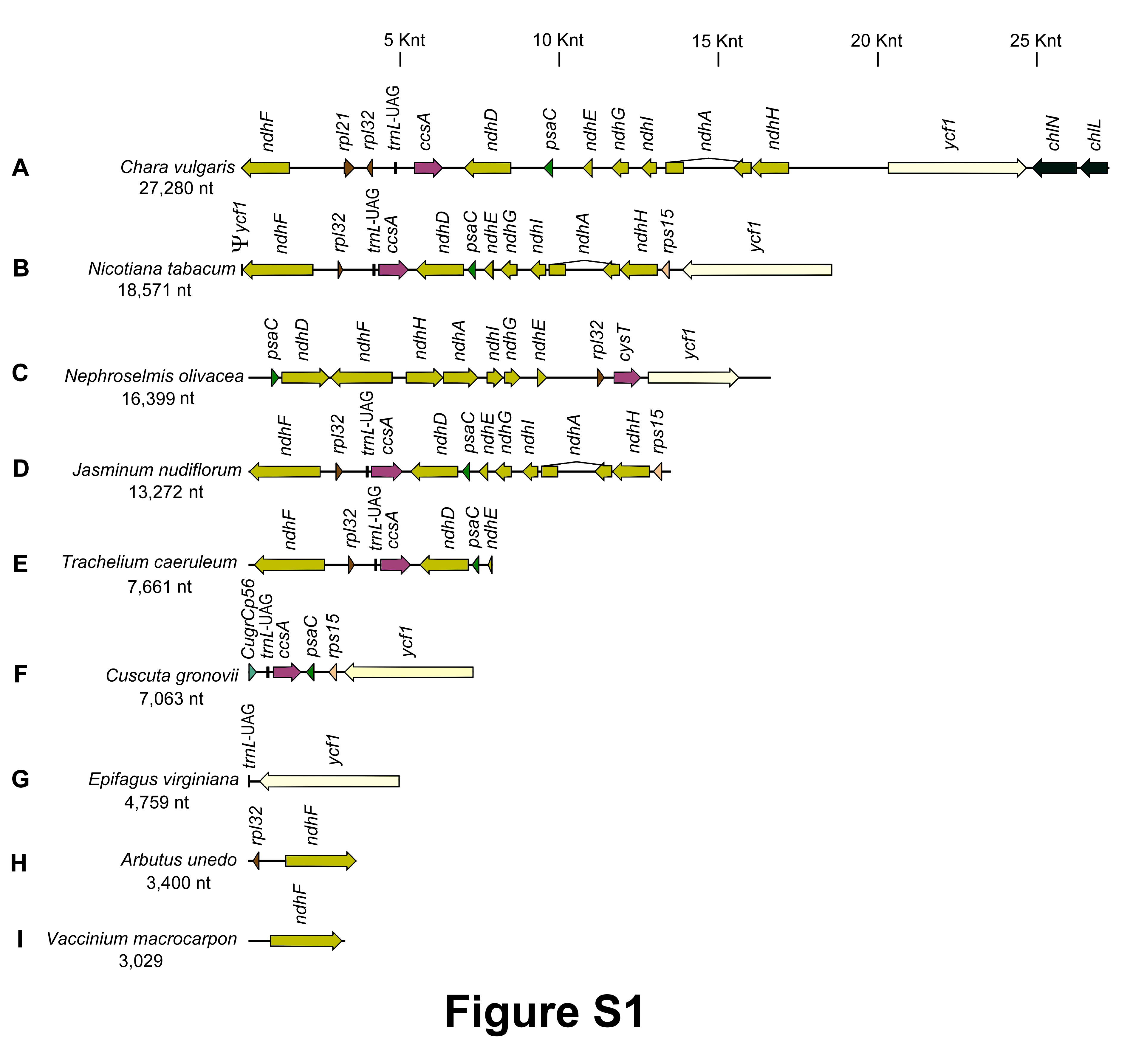

Supplement: Figure S1 — Gene maps representative of the most recurrent variants of the SSC region in plants. Accession numbers of the corresponding genomes are indicated in Table S2. (TIF) [file pone.0079685.s001.tif]

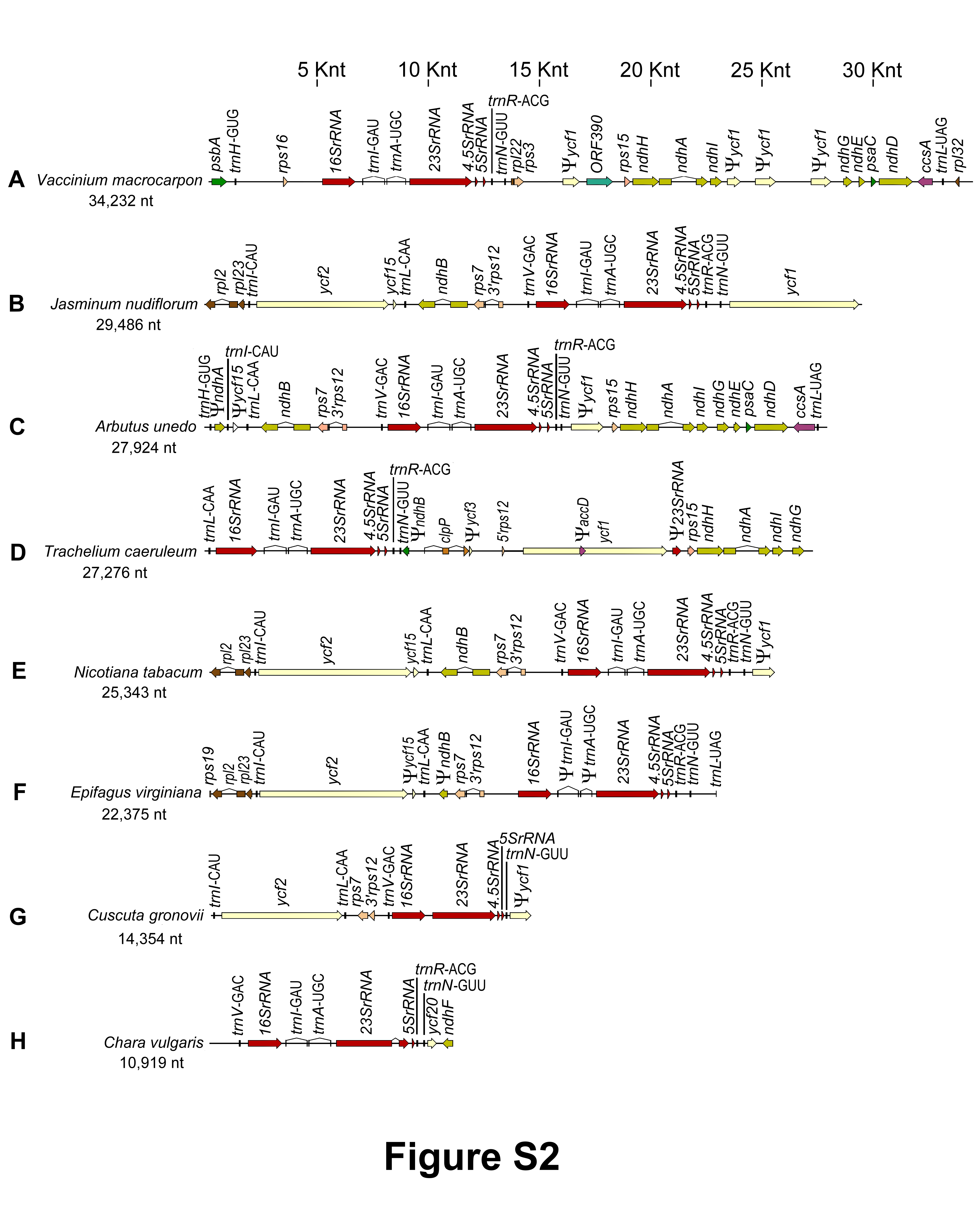

Supplement: Figure S2 — Gene maps representative of the most recurrent variants of the IRs in plants. Accession numbers of the corresponding genomes are indicated in Table S2. (TIF) [file pone.0079685.s002.tif]

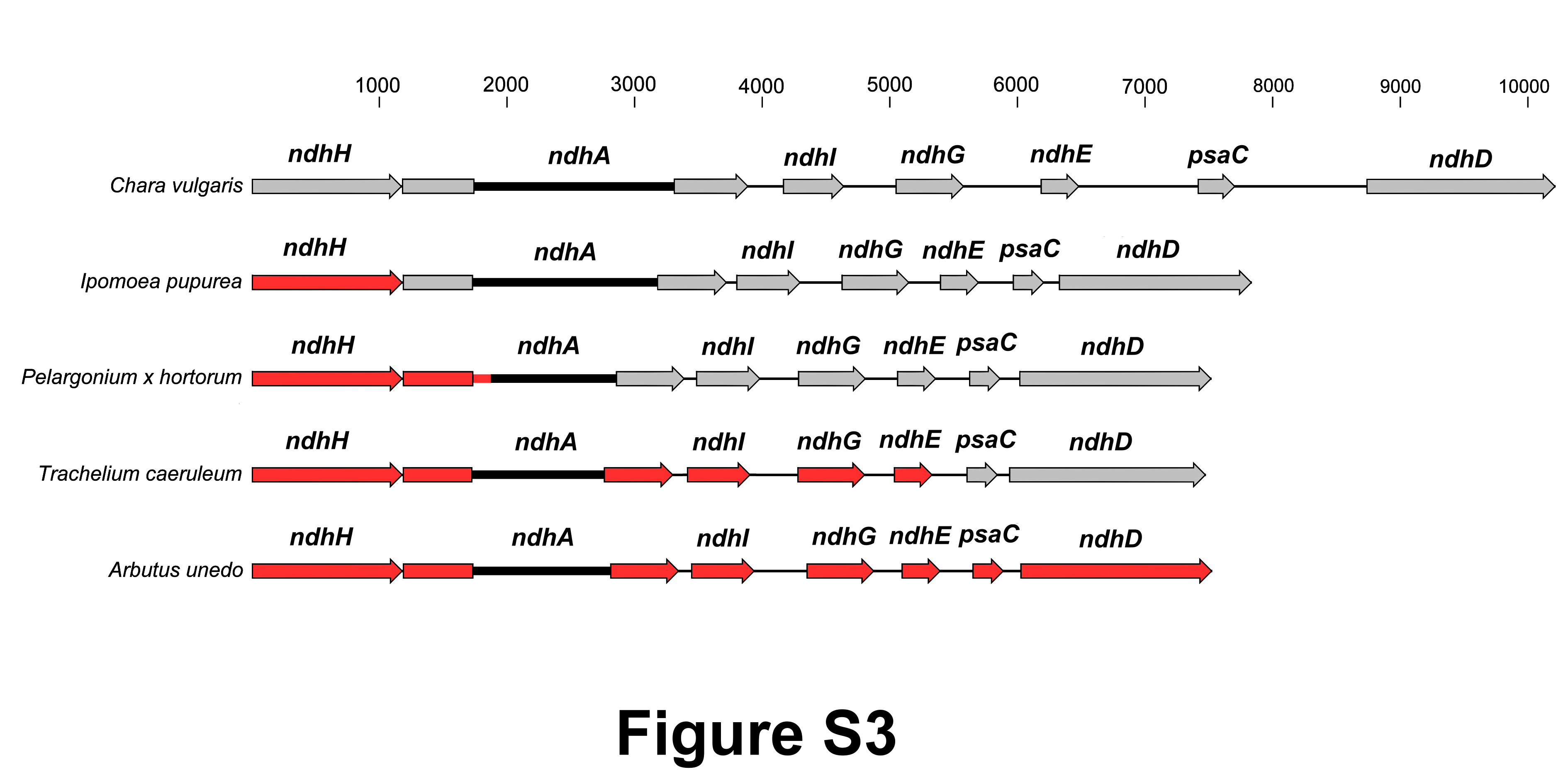

Supplement: Figure S3 — Gene map of the ndhH-D operon in plants showing examples of complete and partial duplications. Coding regions are indicated as arrows. Duplicated portions are indicated in red. Introns and intergenic regions are indicated as thick and thin black bars, respectively. Accession numbers of the corresponding genomes are indicated in Table S2. The scale bar indicates positions in nt. (TIF) [file pone.0079685.s003.tif]

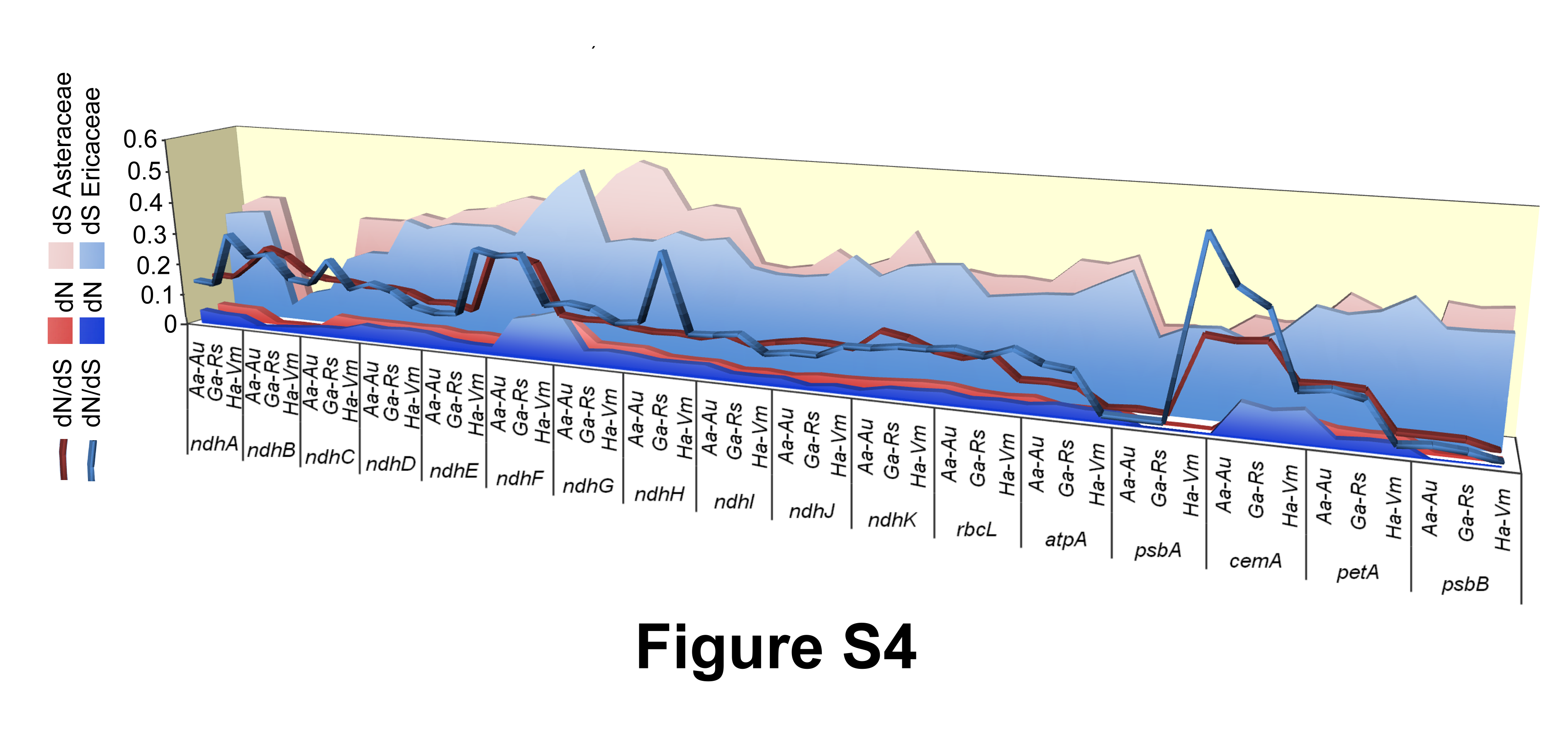

Supplement: Figure S4 — dS and dN values of 17 chloroplast genes. These genes are: ndhA 1017 nt; ndhB 1473 nt; ndhC 342 nt; ndhD 1306 nt; ndhE 303 nt; ndhF 2247 nt; ndhG 396 nt; ndhH 1179 nt; ndhI 487 nt; ndhJ 474 nt; ndhK; 675 nt; rbcL 1425 nt; atpA 1494 nt; psbA 957 nt; cemA 682 nt; petA 963 nt; psbB 1515 nt. Diagram shows the pairwise dS values and dN values along with the dN/dS values between six asterid species and the outgroup (Gossypium hirsutum), three Ericaceae (Au: Arbutus unedo; Rs: Rhododendron simsii; Vm: Vaccinium macrocarpon) and three Asteraceae (Aa: Ageratina adenophora; Ga: Guizotia abyssinica; Ha: Helianthus annuus). Accession numbers of the corresponding genomes are indicated in Table S2. (TIF) [file pone.0079685.s004.tif]
