# Supplementary material for: Balanced Gene Losses, Duplications and Intensive Rearrangements Led to an Unusual Regularly Sized Genome in Arbutus unedo Chloroplasts
Source: PLoS One. 2013 Nov 18;8(11):e79685. doi: 10.1371/journal.pone.0079685 (PMC3832540; doi:10.1371/journal.pone.0079685)
Supplement: Table S1 — List of primers used to complete gap regions in IR-LSC and IR-SSC junctions. (DOCX) [file pone.0079685.s005.docx]

**Table S1.** List of primers used to complete gap regions in IR-LSC and IR-SSC junctions. Primers marked with asterisks were used in both directions: forward and reverse.

| Primer name | Position | Direction | Sequence (5’ to 3’) |
| --- | --- | --- | --- |
| *psbA* | 473 | R | AACCTTGGTATGGAAGTTATG |
| *2rpoC2 R C* | 19594 | R | CAACTTGAAGTTGCACATTATCCC |
| *IrpoC2 1 F* | 19938 | F | TTAAAGTGATTTGTGTATCTACCCC |
| *IrpoC2 2 R* | 20952 | R | GGCAAAGAGACGAAGAAATAGATT |
| *2rpoC2 F C* | 21320 | F | GGAGAAATGCACTGGAGTACC |
| *6rpoB F* | 25742 | F | CCTATTAATCTGGAAGTTCTT |
| *6rpoB R* | 26801 | R | CCGCATTTAGAAGCCCATTTA |
| *2rpoB R NC* | 30905 | R | AACACGAACCTTCGGCTTCT |
| *2atpE F NC* | 32354 | F | GCTGTTTTGGATCGTGCTACTCCCG |
| *2trnT C* | 57721 | F | TTGAACCGATGACTTACGC |
| *15atpE* | 59576 | F | ACGCCGGTCGAGAGAAAG |
| *16atpE* | 60444 | F | ATTCGAGTCTGAGATAGACGC |
| *6accD F* | 60315 | F | TAGTTCAGATAGAATCGACCTT |
| *5AccD R C* | 61813 | R | TTATTTTGCCTATGCCTGTTTGAA |
| *2AccD R NC* | 61965 | R | CACCCCCAGAAGCGCACACT |
| *rpl2* | 94029 | F | CGGGTTAGAAGTTTTGTATAA |
| *A7 90192 F* | 95100 | F | CTGTAATAGCGATGGTATC |
| *trnI.CAU* | 95498 | F | GTTCAATTCCTACTGGATGCA |
| *ycf15* | 95786 | R | CTAAGGTCCAAAATATGGAAG |
| *6rps12 F* | 100798 | F/R* | GGGGGAAGGGTTAAGGATTTA |
| *7rps12 3end F* | 100819 | F/R* | TAAATCCTTAACCCTTCCCCC |
| *6rps15 F* | 110670 | F/R* | ACGGATTTTTGTCTCTCGAA |
| *7rps15 F* | 113160 | F/R* | CGTTGACGTTTTCCCAGAATTTT |
| *13ccsA F* | 121511 | F | TTTCGGGAGCAGTATGGGC |
| *A7 110249 F* | 122818 | F | GAGAATTCAGTAAACAGAAGA |
| *6rpl32 F* | 123248 | F | TCGAGTTGGATGTGAAAGACATC |
| *A7 110674 R* | 123268 | R | TCTGTTTGAATAATAGATGTC |
| *6ndhF R* | 124129 | R | CACAGGAACGTAAAGTGGAACAA |
| *7ndhF R* | 124299 | R | GAYCCAAGACCATACATATTGATA |
| *14ndhF R* | 125822 | R | TCCTTTCAATCAAGCGGGAATG |
| *13ndhF R* | 126109 | R | GTCAAATAATCGTGGTTACATAGATG |
| *A7 110249 F* | 126378 | F | GAGAATTCAGTAAACAGAAGA |
| *11ndhF F* | 126637 | F | AGACGAAAGGTTGAAGTTAAGTAAA |
| *13ccsA F* | 127683 | F/R* | TTTCGGGAGCAGTATGGGC |
| *6rps15* | 113251 | F/R* | ACGGATTTTTGTCTCTCGAA |
| *6rps12 F* | 148591 | F/R* | GGGGGAAGGGTTAAGGATTTA |
| *7rps12 3end F* | 148612 | F/R* | TAAATCCTTAACCCTTCCCCC |
| *trnI.CAU* | 153931 | F | GTTCAATTCCTACTGGATGCA |
