# Supplementary material for: Balanced Gene Losses, Duplications and Intensive Rearrangements Led to an Unusual Regularly Sized Genome in Arbutus unedo Chloroplasts
Source: PLoS One. 2013 Nov 18;8(11):e79685. doi: 10.1371/journal.pone.0079685 (PMC3832540; doi:10.1371/journal.pone.0079685)
Supplement: Table S2 — List of taxa included in either text or figures with GenBank accession numbers and the corresponding bibliographic references. (DOCX) [file pone.0079685.s006.docx]

**Table S2** List of taxa included in either text or figures with GenBank accession numbers, which include the referred sequences, either of the complete genome or specific parts. References are given following the table.

| **Taxon** | **GenBank Accessions** | **Reference** |
| --- | --- | --- |
| **Chlorophyta** |  |  |
| *Chlamydomonas reinhardtii* | NC_005353 | [1] |
| *Chlorella vulgaris* | NC_001865 | [2] |
| *Nephroselmis olivacea* | NC_000927 | [3] |
| *Oocystis solitaria* | FJ968739 | [4] |
| *Ostreococcus tauri* | NC_008289 | [5] |
| **Streptophyta** |  |  |
| **Streptophytina** |  |  |
| *Chara vulgaris* | NC_008097 | [6] |
| *Chaetosphaeridium globosum* | NC_004115 | [7] |
| *Chlorokybus atmophyticus* | NC_008822 | [8] |
| *Mesostigma viride* | NC_002186 | [9] |
| **Spermatophyta** |  |  |
| **Coniferophyta** |  |  |
| *Cephalotaxus wilsoniana* | NC_016063 | [10] |
| *Cryptomeria japonica* | NC_010548 | [11] |
| *Taiwania cryptomerioides* | NC_016065 | [10] |
| **Core Eudicotyledons** |  |  |
| **Asterids** |  |  |
| **Cornales** |  |  |
| *Cornus florida* | EU002157, EU002175, EU002215, EU002276, EU002311, EU002377, EU002491, GQ998074-GQ998146 | [12]  [13] |
| *Davidia involucrata* | GU226265, HM100286-HM100288 | (Li and Zhang, 2011)* |
|  |  | [14] |
| **Ericales** |  |  |
| *Arbutus unedo* | JQ067650 | This study |
| *Ardisia polysticta* | NC_021121 | [15] |
| *Camellia sinensis* | NC_020019 | (Hong *et al*., 2012)* |
| *Franklinia alatamaha* | HM100287-HM100423 | (Li and Zhang, 2011)* |
| *Rhododendron simsii* | GQ997782 -GQ997859 | [13] |
| *Vacccinium macrocarpon* | NC_019616 | [16] |
| **Campanulids** |  |  |
| **Apiales** |  |  |
| *Anethum graveolens* | EU016721-EU016801 | [17] |
| *Anthriscus cerefolium* | NC_015113 | (Downie S.R., 2011)* |
| *Crithmum maritimum* | NC_015804 | (Downie S.R., 2011)* |
| *Daucus carota* | NC_008325 | [18] |
| *Eleutherococcus senticosus* | NC_016430 | (Yi *et al*., 2011)* |
| *Hydrocotyle sp* | NC_015818 | (Downie S.R., 2011)* |
| *Panax ginseng* | NC_006290 | [19] |
| *Petroselinum crispum* | NC_015821  DEFINITION Petroselinum crispum chloroplast, complete genome.  ACCESSION NC_015821  VERSION NC_015821.1 GI:340034260 | (Downie S.R., 2011)* |
| **Aquifoliales** |  |  |
| *Ilex cornuta* | GQ997298-GQ997380 | [13] |
| **Asterales** |  |  |
| *Ageratina adenophora* | NC_015621 | [20] |
| *Guizotia abyssinica* | NC_010601  NC_010601 | [21] |
| *Helianthus annuus* | NC_007977 | [22] |
| *Jacobaea vulgaris* | NC_015543 | [23] |
| *Lactuca sativa* | NC_007578 | [22] |
| *Oxypolis greenmanii* | NC_015832 | (Downie S.R., 2011)* |
| *Parthenium argentatum* | NC_013553 | [24] |
| *Scaevola aemula* | EU017139-EU017217 | [17] |
| *Trachelium caeruleum* | NC_010442 | [25] |
| **Dipsacales** |  |  |
| *Lonicera japonica* | GQ997381-GQ997463 | [13] |
| **Lamiids** |  |  |
| **Boraginaceae (incertae sedis)** |  |  |
| *Ehretia acuminata* | GQ997215-GQ997297 | [13] |
| **Garryales** |  |  |
| *Aucuba japonica* | GQ997049-GQ997131 | [13] |
| **Gentianales** |  |  |
| *Asclepias syriaca* | JF433943 | [26] |
| *Coffea arabica* | NC_008535 | [27] |
| *Nerium oleander* | GQ997630-GQ997712 | [13] |
| **Lamiales** |  |  |
| *Boea hygrometrica* | NC_016468 | [28] |
| *Epifagus virginiana* | NC_001568 | [29] |
| *Jasminum nudiflorum* | NC_008407 | [30] |
| *Olea europea* | NC_013707 | [31] |
| *Olea europea subsp. cuspidata* | NC_015604 | [31] |
| *Olea europea subsp. europaea* | NC_015401 | [31] |
| *Olea europea subsp. maroccana* | NC_015623 | [31] |
| *Olea woodiana subsp. woodiana* | NC_015608 | [31] |
| *Sesamum indicum* | NC_016433 | [32] |
| *Tectona grandis* | NC_020098 | (Volkaert, 2013)* |
| **Solanales** |  |  |
| *Atropa belladonna* | NC_004561 | [33] |
| *Capsicum annuum* | NC_018552 | [34] |
| *Cuscuta exalta* | NC_009963 | [35] |
| *Cuscuta gronovii* | NC_009765 | [36] |
| Cuscuta obtusiflora | NC_009949 | [35] |
| *Cuscuta reflexa* | NC_009766 | [36] |
| *Datura stramonium* | NC_018117 | (Li, *et al.,* 2012)* |
| *Ipomoea purpurea* | NC_009808 | [35] |
| *Nicotiana sylvestris* | NC_007500 | [37] |
| *Nicotiana tabacum* | NC_001879 | [38] |
| *Nicotiana tomentosiformis* | NC_007602 | [37] |
| *Nicotiana undulata* | NC_016068 | [39] |
| *Solanum bulbocastanum* | NC_007943 | [40] |
| *Solanum lycopersicum* | NC_007898 | [41] |
| *Solanum tuberosum* | NC_008096 | [42] |
| **Rosids** |  |  |
| **Malvids** |  |  |
| **Geraniales** |  |  |
| *Monsonia speciosa* | NC_014582 | [43] |
| *Pelargonium x hortorum* | NC_008454 | [44] |
| **Fabids** |  |  |
| **Fabales** |  |  |
| *Cicer arietinum* | NC_011163 | [17] |
| *Medicago truncatula* | NC_003119 | (Lin,S. *et al*., 2009)* |
| *Phaseolus vulgaris* | NC_009259 | [45] |
| *Vigna radiata* | NC_013843 | [46] |
| **Malpighiales** |  |  |
| *Populus alba* | NC_008235 | [47] |
| *Populus trichocarpa* | NC_009143 | [48] |
| **Malvales** |  |  |
| *Gossypium hirsutum* | NC_007944 | [49] |

* Unpublished

**References**

1. Maul JE, Lilly JW, Cui L, DePamphilis CW, Miller W, et al. (2002) The *Chlamydomonas reinhardtii* plastid chromosome: islands of genes in a sea of repeats. Plant Cell 14: 2659-2679.
2. Wakasugi T, Nagai T, Kapoor M, Sugita M, Ito M, et al. (1997) Complete nucleotide sequence of the chloroplast genome from the green alga *Chlorella vulgaris*: the existence of genes possibly involved in chloroplast division. Proc Natl Acad Sci U S A 94: 5967-5972.
3. Turmel M, Otis C, Lemieux C (1999) The complete chloroplast DNA sequence of the green alga *Nephroselmis olivacea*: insights into the architecture of ancestral chloroplast genomes. Proc Natl Acad Sci USA 96: 10248-10253.
4. Turmel M, Gagnon MC, O'Kelly CJ, Otis C, Lemieux C (2009) The chloroplast genomes of the green algae *Pyramimonas*, *Monomastix*, and *Pycnococcus* shed new light on the evolutionary history of prasinophytes and the origin of the secondary chloroplasts of euglenids. Mol Biol Evol 26: 631-648.
5. Robbens S, Derelle E, Ferraz C, Wuyts J, Moreau H, et al. (2007) The complete chloroplast and mitochondrial DNA sequence of *Ostreococcus tauri*: organelle genomes of the smallest eukaryote are examples of compaction. Mol Biol Evol 24: 956-968.
6. Turmel M, Otis C, Lemieux C (2006) The chloroplast genome sequence of *Chara vulgaris* sheds new light into the closest green algal relatives of land plants. Mol Biol Evol 23: 1324-1338.
7. Turmel M, Otis C, Lemieux C (2002) The chloroplast and mitochondrial genome sequences of the charophyte *Chaetosphaeridium globosum*: insights into the timing of the events that restructured organelle DNAs within the green algal lineage that led to land plants. Proc Natl Acad Sci U S A 99: 11275-11280.
8. Lemieux C, Otis C, Turmel M (2007) A clade uniting the green algae *Mesostigma viride* and *Chlorokybus atmophyticus* represents the deepest branch of the Streptophyta in chloroplast genome-based phylogenies. BMC Biol 5: 2.
9. Lemieux C, Otis C, Turmel M (2000) Ancestral chloroplast genome in *Mesostigma viride* reveals an early branch of green plant evolution. Nature 403: 649-652.
10. Wu CS, Wang YN, Hsu CY, Lin CP, Chaw SM (2011) Loss of different inverted repeat copies from the chloroplast genomes of Pinaceae and cupressophytes and influence of heterotachy on the evaluation of gymnosperm phylogeny. Genome Biol Evol 3: 1284-1295.
11. Hirao T, Watanabe A, Kurita M, Kondo T, Takata K (2008) Complete nucleotide sequence of the *Cryptomeria japonica* D. Don. chloroplast genome and comparative chloroplast genomics: diversified genomic structure of coniferous species. BMC Plant Biol 8: 70-2229-8-70.
12. Wang H, Moore MJ, Soltis PS, Bell CD, Brockington SF, et al. (2009) Rosid radiation and the rapid rise of angiosperm-dominated forests. Proc Natl Acad Sci U S A 106: 3853-3858.
13. Moore MJ, Soltis PS, Bell CD, Burleigh JG, Soltis DE (2010) Phylogenetic analysis of 83 plastid genes further resolves the early diversification of eudicots. Proc Natl Acad Sci U S A 107: 4623-4628.
14. Schenk JJ, Hufford L (2010) Effects of substitution models on divergence time estimates: simulations and an empirical study of model uncertainty using Cornales. Systematic Botany 35: 578-592.
15. Ku C, Hu JM, Kuo CH (2013) Complete plastid genome sequence of the basal Asterid *Ardisia polysticta* Miq. and comparative analyses of asterid plastid genomes. PLoS One 8: e62548.
16. Fajardo D, Senalik D, Ames M, Zhu H, Steffan SA, et al. (2013) Complete plastid genome sequence of *Vaccinium macrocarpon*: structure, gene content, and rearrangements revealed by next generation sequencing. Tree Genet Genomes 9: 489-498.
17. Jansen RK, Wojciechowski MF, Sanniyasi E, Lee SB, Daniell H (2008) Complete plastid genome sequence of the chickpea (*Cicer arietinum*) and the phylogenetic distribution of *rps12* and *clpP* intron losses among legumes (Leguminosae). Mol Phylogenet Evol 48: 1204-1217.
18. Ruhlman T, Lee SB, Jansen RK, Hostetler JB, Tallon LJ, et al. (2006) Complete plastid genome sequence of *Daucus carota*: implications for biotechnology and phylogeny of angiosperms. BMC Genomics 7: 222.
19. Kim KJ, Lee HL (2004) Complete chloroplast genome sequences from Korean ginseng (*Panax schinseng* Nees) and comparative analysis of sequence evolution among 17 vascular plants. DNA Res 11: 247-261.
20. Nie X, Lv S, Zhang Y, Du X, Wang L, et al. (2012) Complete chloroplast genome sequence of a major invasive species, crofton weed (*Ageratina adenophora*). PLoS One 7: e36869.
21. Dempewolf H, Kane NC, Ostevik KL, Geleta M, Barker MS, et al. (2010) Establishing genomic tools and resources for *Guizotia abyssinica* (L.f.) Cass.- the development of a library of expressed sequence tags, microsatellite loci, and the sequencing of its chloroplast genome. Mol Ecol Resour 10: 1048-1058.
22. Timme RE, Kuehl JV, Boore JL, Jansen RK (2007) A comparative analysis of the *Lactuca* and *Helianthus* (Asteraceae) plastid genomes: identification of divergent regions and categorization of shared repeats. Am J Bot 94: 302-312.
23. Doorduin L, Gravendeel B, Lammers Y, Ariyurek Y, Chin-A-Woeng T, et al. (2011) The complete chloroplast genome of 17 individuals of pest species *Jacobaea vulgaris*: SNPs, microsatellites and barcoding markers for population and phylogenetic studies. DNA Res 18: 93-105.
24. Kumar S, Hahn FM, McMahan CM, Cornish K, Whalen MC (2009) Comparative analysis of the complete sequence of the plastid genome of *Parthenium argentatum* and identification of DNA barcodes to differentiate *Parthenium* species and lines. BMC Plant Biol 9: 131-2229-9-131.
25. Haberle RC, Fourcade HM, Boore JL, Jansen RK (2008) Extensive rearrangements in the chloroplast genome of *Trachelium caeruleum* are associated with repeats and tRNA genes. J Mol Evol 66: 350-361.
26. Straub SC, Fishbein M, Livshultz T, Foster Z, Parks M, et al. (2011) Building a model: developing genomic resources for common milkweed (*Asclepias syriaca*) with low coverage genome sequencing. BMC Genomics 12: 211-2164-12-211.
27. Samson N, Bausher MG, Lee SB, Jansen RK, Daniell H (2007) The complete nucleotide sequence of the coffee (*Coffea arabica* L.) chloroplast genome: organization and implications for biotechnology and phylogenetic relationships amongst angiosperms. Plant Biotechnol J 5: 339-353.
28. Zhang T, Zhang X, Hu S, Yu J (2011) An efficient procedure for plant organellar genome assembly, based on whole genome data from the 454 GS FLX sequencing platform. Plant Methods 7: 38-4811-7-38.
29. Wolfe KH, Morden CW, Palmer JD (1992) Function and evolution of a minimal plastid genome from a nonphotosynthetic parasitic plant. Proc Natl Acad Sci USA 89: 10648-10652.
30. Lee HL, Jansen RK, Chumley TW, Kim KJ (2007) Gene relocations within chloroplast genomes of *Jasminum* and *Menodora* (Oleaceae) are due to multiple, overlapping inversions. Mol Biol Evol 24: 1161-1180.
31. Besnard G, Hernandez P, Khadari B, Dorado G, Savolainen V (2011) Genomic profiling of plastid DNA variation in the Mediterranean olive tree. BMC Plant Biol 11: 80-2229-11-80.
32. Yi DK, Kim KJ (2012) Complete chloroplast genome sequences of important oilseed crop *Sesamum indicum* L. PLoS One 7: e35872.
33. Schmitz-Linneweber C, Regel R, Du TG, Hupfer H, Herrmann RG, et al. (2002) The plastid chromosome of *Atropa belladonna* and its comparison with that of *Nicotiana tabacum*: the role of RNA editing in generating divergence in the process of plant speciation. Mol Biol Evol 19: 1602-1612.
34. Jo YD, Park J, Kim J, Song W, Hur CG, et al. (2011) Complete sequencing and comparative analyses of the pepper (*Capsicum annuum* L.) plastome revealed high frequency of tandem repeats and large insertion/deletions on pepper plastome. Plant Cell Rep 30: 217-229.
35. McNeal JR, Kuehl JV, Boore JL, de Pamphilis CW (2007) Complete plastid genome sequences suggest strong selection for retention of photosynthetic genes in the parasitic plant genus *Cuscuta*. BMC Plant Biol 7: 57.
36. Funk HT, Berg S, Krupinska K, Maier UG, Krause K (2007) Complete DNA sequences of the plastid genomes of two parasitic flowering plant species, *Cuscuta reflexa* and *Cuscuta gronovii*. BMC Plant Biol 7: 45.
37. Yukawa M, Tsudzuki T, Sugiura M (2006) The chloroplast genome of *Nicotiana sylvestris* and *Nicotiana tomentosiformis*: complete sequencing confirms that the *Nicotiana sylvestris* progenitor is the maternal genome donor of *Nicotiana tabacum*. Mol Genet Genomics 275: 367-373.
38. Shinozaki K, Ohme M, Tanaka M, Wakasugi T, Hayashida N, et al. (1986) The complete nucleotide sequence of tobacco chloroplast genome: its gene organization and expression. EMBO J 5: 2043-2049.
39. Thyssen G, Svab Z, Maliga P (2012) Cell-to-cell movement of plastids in plants. Proc Natl Acad Sci USA 109: 2439-2443.
40. Daniell H, Lee SB, Grevich J, Saski C, Quesada-Vargas T, et al. (2006) Complete chloroplast genome sequences of *Solanum bulbocastanum*, *Solanum lycopersicum* and comparative analyses with other Solanaceae genomes. Theor Appl Genet 112: 1503-1518.
41. Kahlau S, Aspinall S, Gray JC, Bock R (2006) Sequence of the tomato chloroplast DNA and evolutionary comparison of solanaceous plastid genomes. J Mol Evol 63: 194-207.
42. Chung HJ, Jung JD, Park HW, Kim JH, Cha HW, et al. (2006) The complete chloroplast genome sequences of *Solanum tuberosum* and comparative analysis with Solanaceae species identified the presence of a 241-bp deletion in cultivated potato chloroplast DNA sequence. Plant Cell Rep 25: 1369-1379.
43. Guisinger MM, Kuehl JV, Boore JL, Jansen RK (2011) Extreme reconfiguration of plastid genomes in the angiosperm family Geraniaceae: rearrangements, repeats, and codon usage. Mol Biol Evol 28: 583-600.
44. Chumley TW, Palmer JD, Mower JP, Fourcade HM, Calie PJ, et al. (2006) The complete chloroplast genome sequence of *Pelargonium* x *hortorum*: organization and evolution of the largest and most highly rearranged chloroplast genome of land plants. Mol Biol Evol 23: 2175-2190.
45. Guo X, Castillo-Ramirez S, Gonzalez V, Bustos P, Fernandez-Vazquez JL, et al. (2007) Rapid evolutionary change of common bean (*Phaseolus vulgaris* L) plastome, and the genomic diversification of legume chloroplasts. BMC Genomics 8: 228.
46. Tangphatsornruang S, Sangsrakru D, Chanprasert J, Uthaipaisanwong P, Yoocha T, et al. (2010) The chloroplast genome sequence of mungbean (*Vigna radiata*) determined by high-throughput pyrosequencing: structural organization and phylogenetic relationships. DNA Res 17: 11-22.
47. Okumura S, Sawada M, Park YW, Hayashi T, Shimamura M, et al. (2006) Transformation of poplar (*Populus alba*) plastids and expression of foreign proteins in tree chloroplasts. Transgenic Res 15: 637-646.
48. Tuskan GA, Difazio S, Jansson S, Bohlmann J, Grigoriev I, et al. (2006) The genome of black cottonwood, *Populus trichocarpa* (Torr. & Gray). Science 313: 1596-1604.
49. Lee SB, Kaittanis C, Jansen RK, Hostetler JB, Tallon LJ, et al. (2006) The complete chloroplast genome sequence of *Gossypium hirsutum*: organization and phylogenetic relationships to other angiosperms. BMC Genomics 7: 61.
